# Premature Birth Infants Present Elevated Inflammatory Markers in the Meconium

**DOI:** 10.3389/fped.2020.627475

**Published:** 2021-01-18

**Authors:** María Victoria Rodríguez-Benítez, Reyes Gámez-Belmonte, Mercedes Gil-Campos, Cristina Hernández-Chirlaque, Paula R. Bouzas, Fermín Sánchez de Medina, Olga Martínez-Augustin

**Affiliations:** ^1^Unit of Neonatology, Reina Sofía University Hospital, IMIBIC, Córdoba, Spain; ^2^Department of Pharmacology, CIBERehd, School of Pharmacy, Instituto de Investigación Biosanitaria ibs.GRANADA, University of Granada, Granada, Spain; ^3^Unit of Pediatrics Metabolism, Reina Sofia University Hospital, University of Córdoba, IMIBIC, CIBEROBN, Córdoba, Spain; ^4^Department of Biochemistry and Molecular Biology II, CIBERehd, School of Pharmacy, Instituto de Investigación Biosanitaria ibs.GRANADA, Instituto de Ciencia y Tecnología de los Alimentos José Mataix, University of Granada, Granada, Spain; ^5^Department of Statistics, University of Granada, Granada, Spain

**Keywords:** intestinal inflammation, preterm newborns, birth weight, gestational age, neutrophils

## Abstract

**Introduction:** Prematurity, a well-established risk factor for various intestinal diseases in newborns, results in increased morbidity and mortality. However, the intestinal inflammatory status of preterm (PT) infants has been poorly characterized. Here we have broadly described the intestinal and systemic inflammatory status of PT children.

**Materials and Methods:** Meconium and plasma from 39 PT and 32 full term (T) newborns were studied. Fecal calprotectin, polymorphonuclear leukocyte elastase (PMN-E), TNF, IL-17A, IL-8, IP-10, MCP-1, MIP-1, IL-1β, IL-1α, and E-selectin and the enzymatic activities of myeloperoxidase (MPO) and alkaline phosphatase (AP) in meconium were measured. Plasma levels of AP, hepatocyte growth factor, nerve growth factor (NGF), proinflammatory cytokines, leptin, adiponectin, PAI-1, and resistin were also determined. Correlations with gestational age (GA) and birth weight (BW) were studied.

**Results:** Neutrophil derived PMN-E, MPO and calprotectin were increased in the meconium of PT compared to T newborns, while AP was decreased. No significant differences were found in other inflammatory parameters. Considering data from all children, GA and BW showed inverse correlation with neutrophil markers, while AP directly correlated with BW. Plasma levels of IL-1β and NGF were enhanced in PT infants, and were also negatively correlated with BW. PT children additionally showed neutropenia and decreased adiponectin, leptin, haematocrit, and haemoglobin. These parameters (neutrophils, adiponectin, and so forth) were positively correlated with GA and BW, while IL-8, MCP-1, PAI-1, and plasma AP were negatively correlated. PT children showing postnatal morbidity exhibited increased meconium MPO and MIP-1α.

**Conclusion:** PT neonates present a significant elevation of intestinal inflammatory parameters, characterized by the presence of neutrophil markers, associated with mild systemic inflammation.

## Introduction

Preterm (PT) birth is the leading cause of neonatal mortality and morbidity worldwide (1). It has been related to intestinal and immune immaturity. Neonatal meconium, the first stool of a newborn, accumulates proteins naturally excreted by the neonate, reflecting not only the neonate status but also the influence of intrauterine and overall maternal environment (2). Therefore, the characterization of inflammatory markers in meconium is a valuable source of information on the state of the intestine of the newborn, and should relate to the maturation of both the intestine and the immune system. Neutrophil derived proteins like calprotectin, PMN-Elastase (PMN-E), lactoferrin, or myeloperoxidase (MPO) have been shown to be increased in meconium of newborn infants (3). Among them, fecal calprotectin has been proposed as a marker of intestinal inflammation (3–5) and a predictor in the diagnosis of necrotizing enterocolitis, a severe inflammatory condition, in PT newborns (6). Although fecal calprotectin has been measured in feces of PT, only a few studies have specifically assessed it in the meconium of PT compared to T infants. In this regard, Lagrogia *et al*. reported increased levels of calprotectin in the meconium of preterm infants (7), while a study by Kapel et al. indicated no differences between PT and T neonates (8). Little is known about the levels of other leukocyte derived cytokines. On the other hand, systemic inflammation, in the absence of overt infection, has been described in the first month of life in PT children (9, 10), with enhanced circulating levels of IL-6, IL-12, and granulocyte-colony stimulating factor (G-CSF).

The aim or our study was to characterize and compare intestinal and systemic inflammatory markers in PT and T newborns, and to correlate them with gestational age and weight. Not only neutrophil derived proteins but also other cytokines and inflammatory markers like alkaline phosphatase (AP) have been assessed. In addition, the levels of metabolic hormones such as leptin, adiponectin, or resistin have been studied.

## Materials and Methods

### Study Design

This is a descriptive, analytical, observational case-control study, which was approved by the Ethical Committee on Human research of the Reina Sofía University Hospital (Córdoba, Spain). Neonates were selected after all inclusion criteria were fulfilled and the informed written consent from the children's legal guardians was obtained. Selection criteria and study groups were as follows. Two groups were established: a group of neonates born ≥37 weeks of gestational age (GA) with birth weight ≥2,500 g (T group), and a group of neonates born before 37 weeks of GA (PT group). Children with any congenital malformation or genetic disease were excluded. Necrotizing enterocolitis was also excluded. Percentile charts of sex and GA at birth were used to assign children to PT and T groups (11). A complete evaluation and physical examination was conducted in all the children. Perinatal data including the occurrence of chorioamnionitis, way of delivery, prenatal corticosteroid administration, APGAR, and fetal pathology were collected. In addition, long term clinical outcome information was gathered (median follow up 9.2 and 4.7 years for PT and T groups, range 4.5–9.7 and 4.4–9.7, respectively).

### Sample Collection and Routine Parameters

Meconium samples were collected in the first 48 h. Blood samples (4 ml) were drawn and divided in several aliquots. Routine parameters including haemoglobin, hematocrit, and alkaline phosphatase (AP) were determined. A blood count was also performed. All samples not used for routine determinations were kept at −80°C until used.

### Fecal Parameters

Calprotectin and PMN-E were measured by ELISA (Immundiagnostik, Bensheim, Germany). MPO and AP activities measured as described before (12). Supernatants from meconium homogenized in hexadecyltrimethylammonium bromide buffer (1:5 w/v) were obtained and TNF, IL-17A, IL-8, interferon γ-induced protein 10 (IP-10), monocyte chemoattractant protein 1 (MCP-1), macrophage inflammatory protein 1 (MIP-1), IL-1β, IL-1α, and E-selectin were assayed using a human multiplex immunoassay (EPX 200-12185-901, eBioscience, San Diego, USA) in a group of randomly selected samples from 22 PT and 27 T infants.

### Plasma Inflammatory Parameters

Milliplex Map Human Adipokine Magnetic Beads Panels HADK1MAG-61K and HADK2MAG-61K from Millipore (Burlington, USA) were used to determine adiponectin, plasminogen activator inhibitor 1 (PAI-1) and resistin, hepatocyte growth factor (HGF), IL-1β, IL-6, IL-8, insulin, leptin, MCP-1, nerve growth factor (NGF), and TNF.

### Sample Size Calculation and Statistical Analysis

For the calculation of group size we focused on fecal calprotectin as a reference, which has a standard deviation of 78 μg/g (7). To achieve a statistical power of 0.8 to detect differences of 60 μg/g at alpha = 0.05 it was calculated that *n* = 28 was required.

All the variables were checked for normality and accordingly parametric or non-parametric tests were carried out. Differences were considered statistically significant when *p*-value (p) was <0.05. The comparison of means test was the most usual hypothesis test needed in this study (parametric *t*-test and non-parametric Mann-Whitney and Kolmogorov-Sminov tests). Once the difference between two variables was assumed, the effect size was assessed by means of the Cohen's d value following the standard interpretation (values close to 0.2, 0.5, or 0.8 meaning a small, medium or large effect). The correlation between variables was studied calculating the Pearson correlation coefficient, r. For follow up clinical outcomes data were compared with Student's *t*-test.

## Results

**Figure 1:**
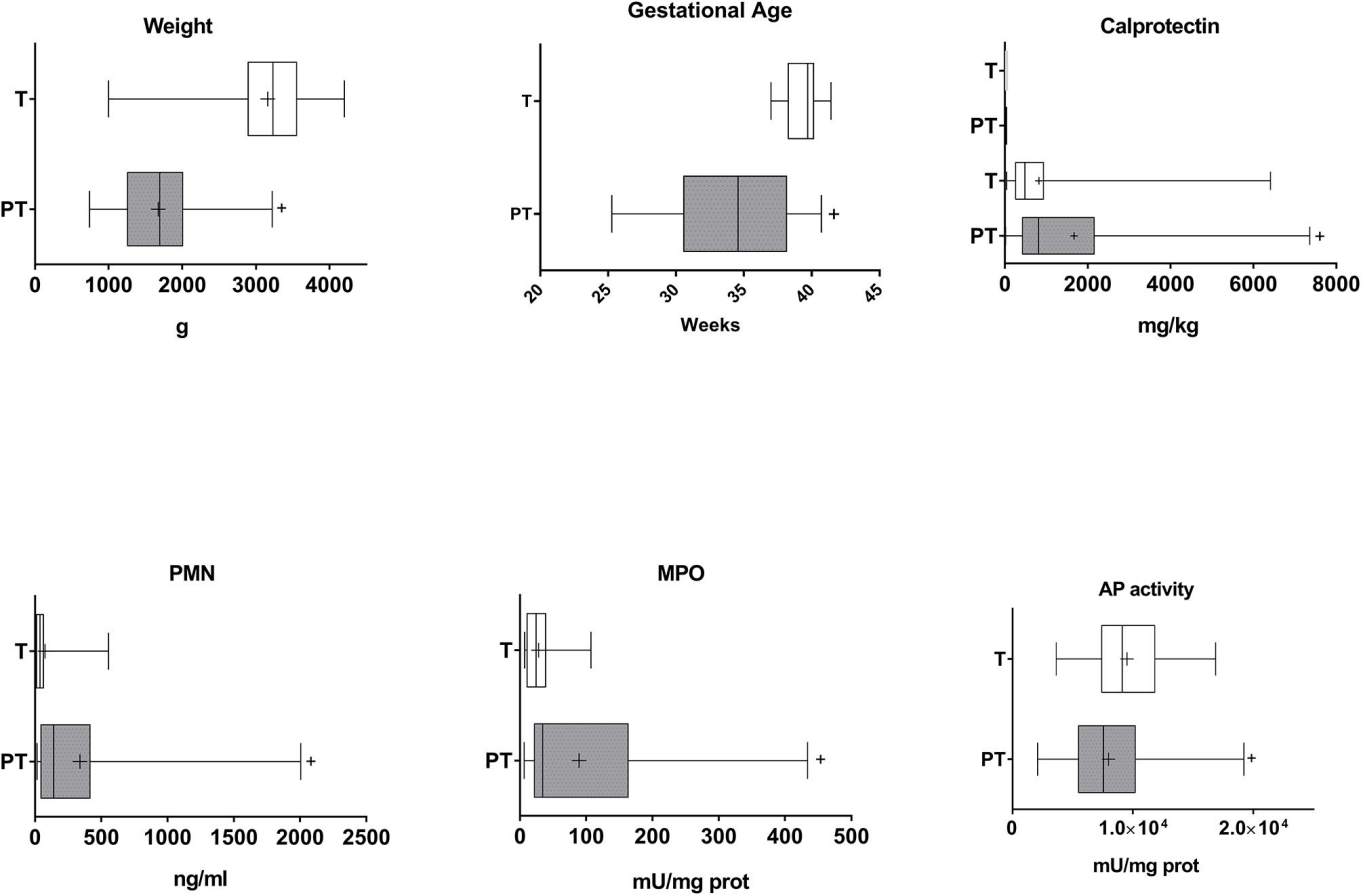
Gestational age, birth weight, and inflammatory markers in the meconium of T and PT infants. Birth weight and gestational age of the newborns and calprotectin, PMN-E, MPO, and AP activity in the feces are shown. ^+^*p* < 0.05 vs. T.

Thirty-two T and 39 PT neonates were enrolled in the study. Only 6 mothers of PT infants (15.4%) presented chorioamnionitis. Eight preterm infants and 18 at term infants were born by cesarean delivery (20.5 and 56.3%, respectively). APGAR for preterm infants was lower than that of T infants (mean ± SEM: 6.8 ± 0.3 vs. 8.4 ± 0.2, *p* < 0.01). Fetal pathology was detected in 6 PT neonates (15.4%), and 23 presented morbidities that include respiratory (*n* = 21, 53.8%), infectious (*n* = 13, 33.3%), neurological (*n* = 9, 23.1%) and haemodynamic/circulatory (*n* = 9, 23.1%) involvement. As expected body birth weight and gestational age were lower in PT *v*s. T infants (Figure 1). Both parameters showed a strong correlation in PT children (Supplementary Table 1) while correlation was weaker in T children. A correlation between body weight and gestational age was also observed when data from both groups were studied together (*r* = 0.817, *p* = 0.000).

### Preterm Infants Present Mild Intestinal Inflammation

**Figure 2:**
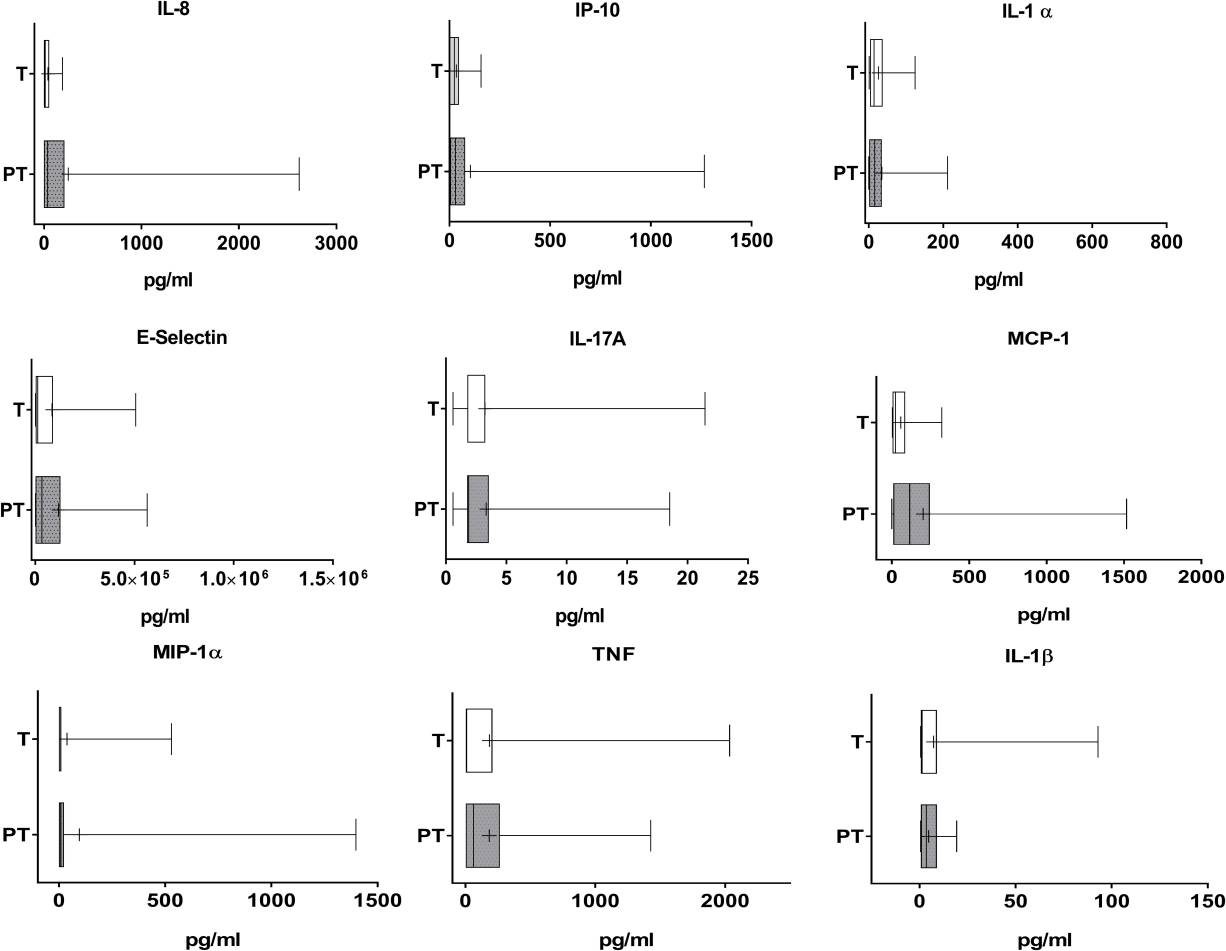
Cytokine and adhesion molecules levels in the meconium of T and PT newborns. IL-8, IP-10, IL-1α, E-selectin, IL-17A, MCP-1, MIP-1α, TNF, and IL-1β are shown. ^+^*p* < 0.05 vs. T.

PT meconium showed a large and medium increase in PMN-E and MPO compared with T infant levels (Figure 1, Cohen's d values 0.773 and 0.369, respectively). Calprotectin was also increased, with a medium effect (Figure 1, Cohen's d value = 0.521). All the other inflammatory markers studied were detected in meconium, although at very low levels in both groups, and no differences were found (Figure 2). AP activity was moderately decreased in the meconium of PT infants (Figure 1, Cohen's d value = 0.473). Of note, levamisole inhibited AP activity substantially (more than 99%), indicating that intestinal alkaline phosphatase was not the predominant isoform present in feces.

In accordance with the notion that gestational age and weight are related to intestinal inflammation, when PT and T newborns were analyzed together, gestational age and/or weight negatively correlated with meconium MPO, calprotectin and PMN-E levels (Table 1). In turn, AP positively correlated with birth weight. PT infants showed a slight positive correlation between body weight and meconium IL-1β (Supplementary Table 1). As for T newborns, gestational age was slightly and positively correlated with both meconium IL-1α and E-selectin and little and negatively with calprotectin and PMN-E (Supplementary Table 1).

**Table 1:** Correlations of gestational age and birth weight with meconium and plasma parameters.

		**Gestational age**		**Birth weight**	
		**Coeficient, *r***	***p*-value**	**Coeficient, *r***	***p*-value**
Meconium	MPO	−0.380	0.002	−0.327	0.007
	Calprotectin	−0.279	0.033	−0.250	0.051
	PMN-E	−0.282	0.023	−0.310	0.010
	AP	n.c	n.c	0.275	0.023
Plasma	IL-8	−0.246	0.049	−0.248	0.042
	MCP1	−0.282	0.023	−0.283	0.050
	AP	−0.351	0.005	−0.319	0.010
	IL-1β	n.c.	n.c.	−0.306	0.011
	NGF	n.c.	n.c.	−0.259	0.033
	PAI-1	−0.256	0.039	n.c.	n.c.
	Haemoglobin	0.387	0.001	0.336	0.004
	Haematocrit	0.474	0.000	0.391	0.001
	Neutrophils	0.743	0.000	0.695	0.000
	Leptin	0.268	0.031	0.345	0.004
	Adiponectin	0.422	0.000	0.371	0.002

*Data from preterm and at term children were analysed together. Only values for statistically significant correlations are shown. Pearson correlation coefficient (r) and p-values are shown. n.c., no correlation*.

Analyzing data from PT and T infants together, we found a correlation between calprotectin and MPO (*r* = 0.363, *p* = 0.044), but not with PMN-E. Strong correlations were generally found among various cytokines (not shown), and between meconium and plasmatic parameters. For instance, meconium MPO was correlated with plasma TNF and adiponectin, haemoglobin, and hematocrit (see Supplementary Table 2 for details).

### Preterm Infants Present Increased Systemic Inflammatory Markers

**Figure 3:**
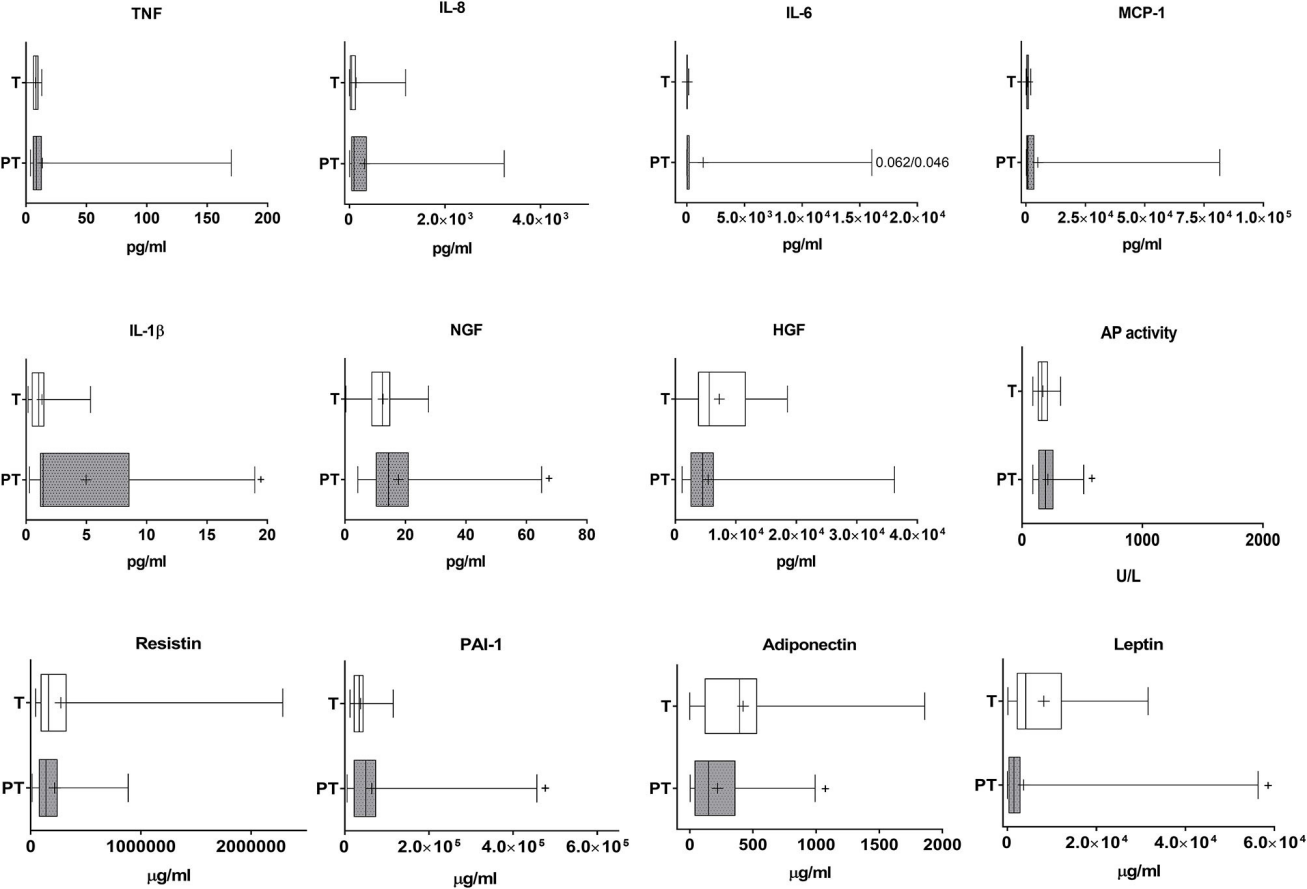
Analysis of cytokines, growth factors, inflammatory factors and haematological markers in the plasma of T and PT newborns. TNF, IL-8, IL-6, MCP-1, IL-1β, NGF, HGF, AP activity, resistin, PAI-1, adiponectin and nectin are shown. ^+^*p* < 0.05 vs. T.

Our data indicate that IL-1β was highly increased in the plasma of PT infants when compared to T newborns (Cohen's d value = 0.931, Figure 3). NGF and AP in serum were also significantly augmented, although differences were very small (Cohens' d values 0.109 and 0.052, respectively, Figure 3). There was no correlation in PT children between gestational age or weight and any of the plasmatic cytokines (Figure 3). In T children a negative slight correlation was found however between gestational age and plasma IL-8 (Supplementary Table 1). When data from all children were considered together, our results indicate that gestational age and weight were negatively and, in general, only slightly correlated to circulating IL-8, MCP-1, and AP. In addition, weight was correlated similarly with IL-1β and NGF and gestational age with PAI-1 (Table 1).

**Figure 4:**
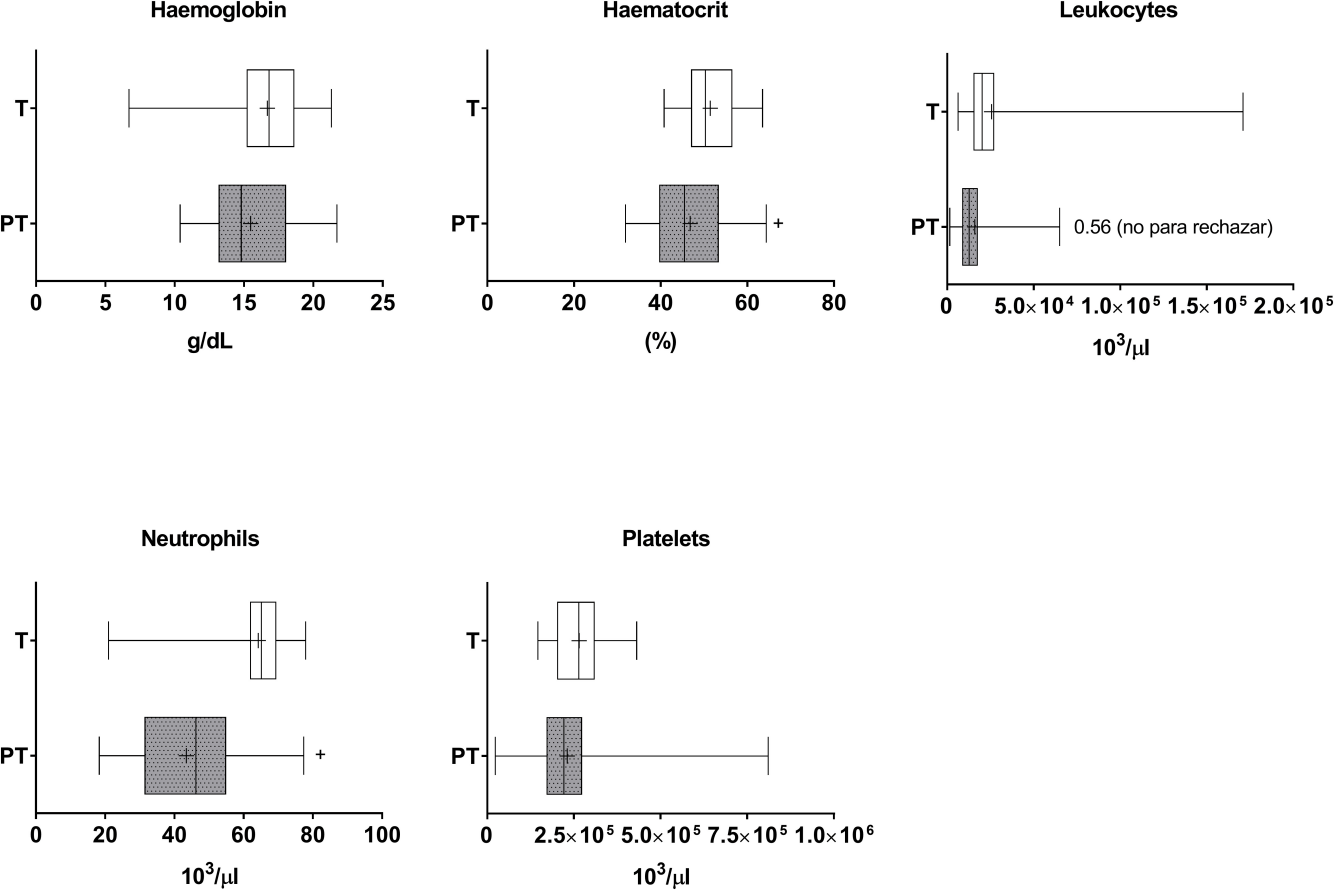
Haematological markers in the plasma of T and PT newborns. Haemoglobin, haematocrit, total leukocytes, neutrophils and platelets are shown. ^+^*p* < 0.05 vs. T.

Although no differences in haemoglobin were found, haematocrit values were decreased in PT when compared to T children, suggesting the presence of anemia (Cohen's d value = 0.618) (Figure 4). Lower neutrophil counts were observed in the blood of PT when compared to T newborns (Cohen's d value = 1.649). The analysis of correlations of the entire cohort (i.e., PT and T newborns) indicated positive albeit very small correlations of gestational age and weight with haemoglobin and hematocrit, and important correlations with neutrophils (Table 1). Positive correlations were similarly found in PT infants (Supplementary Table 1).

### Preterm Children Present Lower Circulating Adiponectin and Leptin Levels

PT children present moderately and importantly lowered levels of circulating leptin and adiponectin (Cohen's d values 0.509 and 1.294, respectively, Figure 3). Both adipokines were positively correlated with gestational age and weight (Table 1).

### Clinical Outcomes

The significant clinical outcomes in the follow up of the newborns is shown in Table 2. As expected, PT exhibited a higher morbidity. Interestingly, the group of PT children with at least one morbidity type exhibited a higher meconium level of MPO activity (139.9 ± 36.9 vs. 63.3 ± 18.4 mU/mg prot, *p* = 0.04) and MIP-1α (273.8 ± 194.2 vs. 20.8 ± 9.0 pg/ml, *p* = 0.04). In addition, calprotectin (2449.0 ± 679.7 vs. 1279.1 ± 305.8 mg/kg, *p* = 0.07) and blood platelets (275,142 ± 46,077 vs. 201,000 ± 15,462, *P* = 0.07) just fell short of significance. No differences were found in the T group.

**Table 2:** Long term morbidities of PT and T children included in the study.

**Morbidities**	**PT**	**T**
Gastrointestinal	3 (GER)	4 (x%) (GER)
Respiratory	4 (asthma/BHR)	2 (asthma/BHR)
Neurological	8 (2 cerebral palsy, 2 autism spectrum, 4 mild maturational delay)	2 (mild maturational delay)
Others	3 (inguinal hernia)	

*GER, Gastroesophageal reflux; BHR, bronchial hyperresponsivenes*.

## Discussion

The immaturity of the intestine and the immune system is a physiological feature of the newborns that, in the case of premature infants, is specially exacerbated. This fact, in combination with factors like diet, microbiota colonization or mode of delivery, has been related to both intestinal and systemic diseases, including necrotizing enterocolitis or allergies. Despite these well-established health risks associated with prematurity, only a few studies have assessed the presence of intestinal inflammation in preterm children, systemic inflammation having received more attention (2, 3). Meconium is a valuable source of information, since proteins accumulated are representative of the intestinal status and, since it is naturally excreted by the neonate, it avoids the need for invasive procedures. In this study, inflammatory biomarkers in meconium samples were measured in PT and T children. Our data suggest that PT children have intestinal inflammation, consistent with an increased presence of neutrophils in meconium, with significantly increased calprotectin, PMN-E and MPO. Fecal calprotectin is widely used as a non-specific marker of intestinal inflammation, particularly in inflammatory bowel disease patients (4), and has been previously studied in PT infants, with sometimes conflictive results, i.e., increased or unchanged levels compared to T infants (7, 8). PMN-E, MPO and calprotectin have been previously shown to be correlated in meconium samples (5). Our results show partial correlation (i.e., calprotectin-MPO but not with PMN-E). To the best of our knowledge this is the first study to assess inflammatory markers such as IL-17, IL-1β, CXCL10, MCP-1, or MIP-1α in PT meconium. The low expression of these markers corroborates the predominance of mild neutrophilic inflammation, i.e., neutrophil infiltration and leakage to the lumen, consistent with acute intestinal inflammation (6, 7). High sensitivity of AP activity is also consistent with intestinal inflammation, as it is characteristic of tissue non-specific rather than intestine type AP (11–13). Enzyme activity itself was actually decreased, as in previous reports (13), probably due to gut immaturity or enzyme instability in an inflammatory context (14). Increased levels of inflammatory parameters (IL-1β and NGF) are present in the plasma of PT infants. Given the nature of our study, these may or may not be related to intestinal inflammation. Even though global statistical analysis showed no additional significant differences, it is worth noting that the data obtained suggest that further effects may be picked up by a study with increased statistical power. The magnitude of the changes in inflammatory markers is of course lower than in frank inflammatory conditions such as necrotizing enterocolitis (8, 15). These findings contrast with the immunological immaturity expected in PT newborns.

As previously described (16–19), we found neutropenia in PT children, correlated with weight and gestational age. This can be secondary to decreased production of neutrophils, increased neutrophil destruction, or a combination of both mechanisms (20), consistent with PT leukopenia. Intestinal accumulation of neutrophils may also contribute, as a secondary transient cause, given the transitory nature of the phenomenon (7–10 days maximum) (18). Marginal redistribution of neutrophils produces neutropenia in NEC and inflammatory bowel disease (21). Other hematological alterations found in our study and previously described (22, 23) include low hematocrit and haemoglobin in PT infants. These anomalies were correlated with gestational age/weight.

We also measured the adipokines leptin, adiponectin, resistin, and PAI-1. Leptin is considered a proinflammatory adipokine, and plasma levels are increased in inflammatory bowel disease (24), while adiponectin is considered anti-inflammatory and regulated negatively in intestinal inflammation. In spite of its adipokine character, resistin in humans is actually produced majoritarily by immune cells (25) and is augmented in chronic colitis (26). In our study, leptin and adiponectin levels were reduced in the plasma of PT infants, while resistin was unchanged. Leptin concentration may be decreased simply as a consequence of low body weight. Adiponectin is negatively correlated with adiposity in adulthood, but not in newborns, suggesting that low plasma levels [consistent with previous reports (27, 28)] may be accounted for by mild inflammation. Contrary to other studies (29, 30), we found similar levels of resistin in PT and T infants. On the other hand, PAI-1 was augmented, consistent with other studies in PT infants (31), as well as in term newborns with low weight (32). Thus, our results indicate a different profile than in T newborns, with uncertain relation with intestinal inflammation. Further studies will be required to explore this possibility.

In order to assess whether our data were correlated in any way with children's postnatal morbidity, we gathered information from the clinical history of the participants. These results should be interpreted with care as the study is of moderate size and was not designed for this purpose. Nevertheless, it is noteworthy that inflammatory parameters in the meconium (MPO, MIP-1α, and calprotectin, the latter just under significance) were increased in PT but not in T infants in which gastrointestinal, respiratory or neurological morbidity occurred. In this regard, recall bias is not expected to influence data collection within the PT group. Hence it is of interest to test in a future, larger study whether such correlation holds and particularly its prognostic potential.

As mentioned above, the composition of meconium is influenced not only by the intestinal status but by the maternal environment. In this regard, it is important to consider the possible impact of chorioamnionitis, a subclinical intrauterine infection and/or inflammation that has been firmly established as a cause of PT delivery (1, 9, 10, 12), as it could partly account for the increased values of inflammatory parameters in PT meconium. In our study, 6 out of 39 PT infants (15.4%) were born to chorioamnionitis diagnosed mothers, 3 of them presenting above average MPO and calprotectin values for this group. When statistical analysis was carried out excluding these 5 samples, differences between the PT and T groups remained significant (data not shown). Thus, chorioamnionitis did not play a major role in the meconium inflammatory profile observed in our cohort.

The findings in our study have several limitations, including the design based on a single time point and the acknowledged influence of the maternal environment on the composition of the meconium, beyond the intestine itself, which may account for the changes detected. The latter are mitigated by the low and balanced occurrence of chorioamnionitis in our cohort, as discussed above.

## Conclusion

PT infants exhibit signs of acute inflammation in the intestinal tract with systemic involvement. Adipokine plasma levels are altered in PT infants, with decreased leptin and adiponectin in PT newborns.

## Data Availability Statement

The original contributions presented in the study are included in the article/Supplementary Materials, further inquiries can be directed to the corresponding author/s.

## Ethics Statement

The studies involving human participants were reviewed and approved by Ethical Committee on Human research of the Reina Sofía University Hospital. Written informed consent to participate in this study was provided by the participants' legal guardian/next of kin.

## Author Contributions

MR-B and MG-C recruited patients and gathered clinical data. RG-B and CH-C carried out the analytical measurements. PB was responsible for statistical analysis. The study was designed by MG-C, FSM, and OM-A. All authors contributed to overall interpretation and analysis and approved the final version of the manuscript.

## Conflict of Interest

The authors declare that the research was conducted in the absence of any commercial or financial relationships that could be construed as a potential conflict of interest.
