# Supplementary material for: Premature Birth Infants Present Elevated Inflammatory Markers in the Meconium
Source: Front Pediatr. 2021 Jan 18;8:627475. doi: 10.3389/fped.2020.627475 (PMC7848191; doi:10.3389/fped.2020.627475)
Supplement: Supplementary file 1 [file Table_1.docx]

Supplementary Table 1: Correlations of gestational age and birth weight with meconium and plasma parameters of preterm (PT) and at term infants (T). Only values for statistically significant correlations are shown. Pearson correlation coefficient (r) and p values are shown. n.c.: no correlation.

|  |  |  | Gestational age | | Birth weight | |
| --- | --- | --- | --- | --- | --- | --- |
|  |  |  | Coefficient, r | p value | Coefficient, r | p value |
| PT |  | Gestational age | - | - | 0.821 | 0.000 |
|  | Meconium | IL-β | n.c. | n.c. | 0.451 | 0.035 |
|  | Plasma | Leptin | 0.268 | 0.031 | 0.345 | 0.004 |
|  |  | Adiponectin | 0.422 | 0.000 | 0.371 | 0.002 |
|  |  | Hemoglobin | 0.387 | 0.000 | 0.336 | 0.004 |
|  |  | Haematocrit | 0.447 | 0.000 | 0.391 | 0.004 |
|  |  | Neutrophils | 0.734 | 0.000 | 0.695 | 0.000 |
| T |  | Gestational age | n.c. | n.c. | 0.340 | 0.035 |
|  | Meconium | IL-1α | 0.474 | 0.023 | n.c. | n.c. |
|  |  | E-selectin | 0.415 | 0.041 | n.c. | n.c. |
|  |  | Calprotectin | -0.282 | 0.023 | n.c. | n.c. |
|  |  | PMN-E | -0.279 | 0.032 | n.c. | n.c. |
|  | Plasma | IL-8 | -0.410 | 0.032 | n.c. | n.c. |
