# Supplementary material for: Premature Birth Infants Present Elevated Inflammatory Markers in the Meconium
Source: Front Pediatr. 2021 Jan 18;8:627475. doi: 10.3389/fped.2020.627475 (PMC7848191; doi:10.3389/fped.2020.627475)
Supplement: Supplementary file 2 [file Table_2.docx]

Supplementary Table 2. Correlations between systemic (blood) and fecal (meconium) parametres. AdpN: adiponectin; CP: calprotectin; Hb: hemoglobin; HC: hematocrit; Leuc: leukocytes; Ne: neutrophils. Pearson correlation coeficient (r) and p value are shown. Correlations with p values >0.05 are not shown.

| Meconium/blood | | AdpN | AP | Hb | HC | HGF | IL-1β | IL-6 | Leuc | Ne | NGF | PCR | TNF |
| --- | --- | --- | --- | --- | --- | --- | --- | --- | --- | --- | --- | --- | --- |
| AP |  |  |  |  |  | 0.432  0.006 |  |  | 0.276  0.023 |  |  |  |  |
| CP |  |  | -0.286  0.018 |  |  |  |  |  |  |  |  |  |  |
| IL-1β |  |  |  |  |  |  |  |  |  |  | 0.339  0.023 |  |  |
| IL8 |  |  | 0.470  0.001 |  |  |  |  |  |  |  |  |  |  |
| IL17A |  |  |  |  |  |  |  | 0.412  0.005 |  |  |  |  |  |
| MCP-1 |  |  | 0.540  0.000 |  |  |  |  |  |  |  |  |  |  |
| MPO |  | -0.250  0.045 |  | -0.314  0.010 | -0.396  0.010 |  |  |  |  | -0.279  0.022 |  | 0.300  0.015 | 0.304  0.014 |
| NGF |  |  |  |  |  |  | 0.339  0.023 |  |  |  |  |  |  |
| PMN |  |  |  | -0.275  0.032 | -0.333  0.009 |  |  |  |  |  |  |  |  |
| TNF |  |  |  |  |  |  |  | 0.358  0.016 |  |  |  |  |  |
